# Non-coding RNAs and Their Roles in Stress Response in Plants

**DOI:** 10.1016/j.gpb.2017.01.007

**Published:** 2017-10-07

**Authors:** Jingjing Wang, Xianwen Meng, Oxana B. Dobrovolskaya, Yuriy L. Orlov, Ming Chen

**Affiliations:** 1 Department of Bioinformatics, State Key Laboratory of Plant Physiology and Biochemistry, College of Life Sciences, Zhejiang University, Hangzhou 310058, China; 2 James D. Watson Institute of Genome Sciences, Zhejiang University, Hangzhou 310058, China; 3 Institute of Cytology and Genetics, Siberian Branch of the Russian Academy of Sciences, Novosibirsk 630090, Russia; 4 Novosibirsk State University, Novosibirsk 630090, Russia

**Keywords:** lncRNA, miRNA, Stress response, RNA-directed DNA methylation, Small RNA

## Abstract

Eukaryotic genomes encode thousands of non-coding RNAs (ncRNAs), which play crucial roles in transcriptional and post-transcriptional regulation of gene expression. Accumulating evidence indicates that ncRNAs, especially microRNAs (miRNAs) and long ncRNAs (lncRNAs), have emerged as key regulatory molecules in plant **stress responses**. In this review, we have summarized the current progress on the understanding of plant **miRNA** and **lncRNA** identification, characteristics, bioinformatics tools, and resources, and provided examples of mechanisms of miRNA- and lncRNA-mediated plant stress tolerance.

## Introduction

Non-coding RNAs (ncRNAs) are functional RNAs with low protein-coding potential. According to their length, ncRNAs can be divided into small ncRNAs (sRNAs) (18–30 nt), medium-sized ncRNAs (31–200 nt), and long ncRNAs (lncRNAs) (>200 nt). Specifically, plant microRNAs (miRNAs) are commonly 21-nt sRNAs, which guide degradation and/or inhibit translation of the mRNA targets, thus suppressing expression of the target genes [1]. In addition to miRNAs, a large number of lncRNAs have been found in plants as well. lncRNAs are originally regarded as transcriptional “noise”, owing to their low levels of expression and sequence conservation compared with protein-coding mRNAs [2], [3], [4], [5]. However, accumulating evidence shows that some lncRNAs execute their functions as *cis*- or *trans*-regulators of gene expression (reviewed in [2], [3], [4], [5]).

In nature, plants are exposed to a wide array of stress factors, such as viral infection, salt, drought, cold, and heat, which limit plant growth and productivity. To adapt and survive under such adverse situations, plants have utilized multiple gene regulatory mechanisms to restore and re-establish cellular homeostasis. Emerging evidence has revealed that ncRNAs play a critical role in the regulation of gene expression in response to stress conditions [6], [7], [8], [9].

With the advantage of the next-generation sequencing technologies and bioinformatics approaches, a great number of ncRNAs have been identified and characterized in plants, especially miRNAs and lncRNAs. In the following sections, we summarize recent knowledge on plant miRNAs and lncRNAs, including their biogenesis, resources, and bioinformatics tools. We also summarize the current status of studies on mechanisms of miRNA- and lncRNA-mediated responses to various stresses, and highlight the key roles of these ncRNAs in response and adaptation to biotic and abiotic stresses.

## Identification, characterization, and collection of ncRNAs in plants

### Plant miRNAs biogenesis

miRNAs, a class of endogenous sRNAs, play an important role in post-transcriptional RNA-mediated silencing of genes. Similar to protein-coding genes, miRNA genes (MIRs) are usually transcribed by RNA polymerase II (RNAPII) [10] to produce primary transcripts (pri-miRNAs). Then pri-miRNAs are produced by DICER-LIKE 1 (DCL1) to generate miRNA–miRNA^∗^ duplexes. Subsequently, these duplexes are methylated by HUA ENHANCER 1 (HEN1) at their 3′ end. Finally, the mature miRNA assembles with ARGONAUTE 1 (AGO1) to form RNA-induced silencing complex (RISC), which specifically targets mRNAs based on sequence complementation, resulting in mRNA degradation and/or translational repression. In addition, although DCL4 primarily functions in small-interfering RNA (siRNA) biogenesis, it has also been found to be involved in biogenesis of a few miRNAs in *Arabidopsis thaliana* [1].

Besides the central components of the plant miRNA biogenesis pathway, DCL1, HYPONASTIC LEAVES1 (HYL1), and SERRATE (SE), some factors have been described to promote pri-miRNA processing through interaction with DCL1 and SE, such as CELL DIVISION CYCLE 5 (CDC5) [11], PLEIOTROPIC REGULATORY LOCUS 1 (PRL1) [12], NEGATIVE ON TATA LESS 2 (NOT2) [13], TOUGH (TGH) [14], and the RNA-binding protein DAWDLE (DDL) [15]. Recent evidence also suggests the key role of elongator in the miRNA biogenesis, as elongator acts as scaffold that recruits DCL1 to the nascent pri-miRNA [16]. These factors, with DCL1, SE, and HYL1, assemble the miRNA processing complex (see the review [17] for the details).

### miRNA identification and target prediction in plants

Generally speaking, miRNAs can be identified by both experimental and computational approaches. However, researchers prefer to apply the computational approaches, as the experimental miRNA identification is typically perceived as complex and time-consuming to implement. Recently, with the advent of high-throughput sequencing, several computational tools have been established to identify and predict miRNAs (Table 1).

**Table 1:** **A summarized list of key tools for identifying and annotating ncRNAs in plants**

**Name**	**Description**	**Web link**	**Ref.**
miRPlant	Identification of miRNAs from RNA-seq data	https://sourceforge.net/projects/mirplant/	[18]

miRanalyzer	Identification of miRNAs and analysis of RNA-seq data	http://bioinfo5.ugr.es/miRanalyzer/miRanalyzer.php	[19]

miRA	Identification of miRNAs in organisms without existing miRNA annotation or without a known related organism with well-characterized miRNAs	https://github.com/mhuttner/miRA	[20]

miRDeep-P	A modified miRDeep to predict plant miRNAs	http://faculty.virginia.edu/lilab/miRDP/	[21]

Semirna	Identification of miRNAs using target sequences	http://www.bioinfocabd.upo.es/semirna/	[22]

TAPIR	Prediction of miRNA targets, including target mimics	http://bioinformatics.psb.ugent.be/webtools/tapir/	[23]

psRNATarget	Prediction of miRNA targets, including reverse complementary matching and target site accessibility evaluation	http://plantgrn.noble.org/psRNATarget/	[24]

miRU	Prediction of miRNA targets using a search algorithm	http://bioinfo3.noble.org/miRU.htm	[25]

MicroPC	Identification of miRNAs and prediction of their targets from large-scale EST analysis	http://www3a.biotec.or.th/micropc/index.html	[26]

C-mii	Identification of miRNAs and prediction of their targets	http://www.biotec.or.th/isl/c-mii	[27]

MTide	Identification of miRNA − target interaction by combining modified miRDeep2 and CleaveLand4	http://bis.zju.edu.cn/MTide/	[28]

PlantMirnaT	Identification of miRNA − target interaction using sRNA-seq data and RNA-seq data	https://sites.google.com/site/biohealthinformaticslab/resources	[29]

BioVLAB-MMIA-NGS	Integrated analysis of miRNAs and mRNAs using high-throughput sequencing data	http://epigenomics.snu.ac.kr/biovlab_mmia_ngs/	[30]

MFSN	Prediction of plant miRNA functions via miRNA − miRNA functional synergistic network		[31]

PhlyoCSF	Calculation of lncRNA coding potential using CSF score	https://github.com/mlin/PhyloCSF	[32]

CPC	Calculation of lncRNA coding potential using sequence features and SVM	http://cpc.cbi.pku.edu.cn/	[33]

CNCI	Calculation of lncRNA coding potential by profiling adjoining nucleotide triplets	https://github.com/www-bioinfo-org/CNCI	[34]

CPAT	Calculation of lncRNA coding potential using a logistic regression model	http://rna-cpat.sourceforge.net/	[35]

DeepLNC	Prediction of lncRNAs using deep neural network	http://bioserver.iiita.ac.in/deeplnc/	[36]

iSeeRNA	Prediction of lncRNAs using SVM algorithm	http://137.189.133.71/iSeeRNA/	[37]

lncRNATargets	Prediction of lncRNA targets based on nucleic acid thermodynamics	http://www.herbbol.org:8001/lrt/index.php	[38]

spongeScan	Identification of miRNA sponges	http://spongescan.rc.ufl.edu/	[39]

TF2LncRNA	Identification of transcription factors	http://mlg.hit.edu.cn/tf2lncrna	[40]

RegRNA	Identification of regulatory RNA motifs	http://regrna2.mbc.nctu.edu.tw/index.html	[41]

*Note*: RNA-seq, RNA sequencing; CSF, codon substitution frequency; SVM, support vector machine.

According to sRNA sequencing (sRNA-seq) data, miRPlant [18], miRanalyzer [19], miRA [20] and miRDeep-P [21] could be used to predict new miRNAs; whereas based on target data, Semirna [22] could be used to search for miRNAs.

A great number of programs and algorithms have been applied for the prediction of miRNA targets. miRU [25], psRNATarget [24], and TAPIR [23] are the three commonly-used tools to predict plant miRNA targets. miRU was designed based on Smith-Waterman algorithm. But unfortunately it is unable to identify the multiplicity of target sites for each target. Instead, psRNATarget was developed based on a parallel iterative Smith-Waterman algorithm and is able to analyze genome-wide high-throughput sRNA-seq data and calculate multiplicity of target sites. Furthermore, TAPIR predicts targets with two modes, “fast” mode and “precise” mode, based on the FASTA search engine and the RNA-hybrid search engine, respectively. Moreover, several integrated bioinformatics tools and software have been developed to identify and predict miRNAs and their targets (Table 1). Taking MTide [28] for example, the combination of a modified miRDeep2 [42], a modified CleaveLand4 [43], and some other useful scripts is used to calculate expression profiles of known MIRs, predict novel miRNAs, and identify target mRNAs. MTide is composed of four modules: miRNA identification with modified miRDeep2, miRNA target identification with modified CleaveLand4, miRNA target prediction with “precise” mode of TAPIR, and prioritization of the predicted target based on GO similarity.

### Databases of plant miRNAs

Several biological databases have been established for archiving miRNAs and their annotation (Table 2).

**Table 2:** **A summarized list of key databases for identifying and annotating ncRNAs in plants**

**Name**	**Description**	**Web link**	**Ref.**
miRBase	miRNAs collected using experimental and computational methods from various species	http://www.mirbase.org/	[44]

Rfam	ncRNA families	http://rfam.sanger.ac.uk/	[45]

DMD	miRNAs from 15 dietary plant and animal species	http://sbbi.unl.edu/dmd/	[46]

PmiRKB	Plant miRNA Knowledge Base	http://bis.zju.edu.cn/pmirkb/	[47]

PMRD	Plant miRNA data information, secondary structure, target genes, and expression profile	http://bioinformatics.cau.edu.cn/PMRD/	[48]

PlanTE-MIR DB	TE-related miRNAs	http://bioinfo-tool.cp.utfpr.edu.br/plantemirdb/	[49]

ASRP	miRNAs, ta-siRNAs, and their targets in Arabidopsis	http://asrp.cgrb.oregonstate.edu/	[50]

miRTarBase	Validated miRNA − target interactions	http://mirtarbase.mbc.nctu.edu.tw/index.php	[51]

miRFANs	miRNA function annotations in Arabidopsis	http://www.cassava-genome.cn/mirfans	[52]

PASmiR	miRNA response to abiotic stress	http://pcsb.ahau.edu.cn:8080/PASmiR	[53]

WMP	miRNA response to abiotic stress in wheat	http://wheat.bioinfo.uqam.ca	[54]

NONCODE	The most complete collection and annotation of ncRNAs from 16 species, including Arabidopsis as the only plant species	http://www.noncode.org/	[55]

GREENC	A wiki-based plant lncRNAs database from 37 plant species	http://greenc.sciencedesigners.com/	[56]

lncRNAdb	Literature describing functions of lncRNAs	http://lncrnadb.org	[57]

PLncDB	lncRNAs from Arabidopsis	http://chualab.rockefeller.edu/gbrowse2/homepage.html	[58]

PNRD	lncRNAs from mainly four plant species, including Arabidopsis, rice, poplar, and maize	http://structuralbiology.cau.edu.cn/PNRD/	[59]

CANTATAdb	lncRNAs from 10 model plant species	http://yeti.amu.edu.pl/CANTATA/	[60]

PLNlncRbase	Experimentally-identified plant lncRNAs	http://bioinformatics.ahau.edu.cn/PLNlncRbase/pcsb	[61]

*Note*: TE, transposable element; ta-siRNA, trans-acting small interfering RNA.

miRBase [44] collects miRNAs from experimental or computational identification of various species, while Rfam [45] provides miRNA sequences based on homology relationship. Other than miRBase and Rfam, plant microRNA database (PMRD) [48] and Plant microRNA Knowledge Base (PmiRKB) [47] are two well-known plant-specific miRNA annotation databases. miRTarBase [51] is the common miRNA–target interaction database. Additionally, two Arabidopsis-specific databases, Arabidopsis Small RNA Project (ASRP) [50] and miRFANs [52], have also been developed, covering miRNA sequences, their targets and related function annotations.

It is worth mentioning that although there are many tools to identify and predict miRNAs and their targets in plants, only two databases contain information related to stress: PASmiR [53] and WMP [54]. PASmiR (http://pcsb.ahau.edu.cn:8080/PASmiR) allows the users to search for miRNA–stress regulatory data in 33 plant species, whereas WMP is for wheat miRNAs only.

### Characteristics of lncRNAs

lncRNAs are longer than 200 nt and usually have low protein-coding potential [62], although *ENOD40* has been reported to encode small peptides [63]. Most lncRNAs are produced by RNAPII, whereas previous work in Arabidopsis identified some lncRNAs transcribed by RNAPIII, which are induced by specific stresses such as hypoxia [64]. Most lncRNAs are polyadenylated [poly(A)+] in plants, however, there are some non-polyadenylated [poly(A)−] lncRNAs as well [65]. In particular, hundreds of poly(A)− lncRNAs were found to be induced by specific abiotic stresses in Arabidopsis [9]. Based on the genomic origins, lncRNAs are broadly divided into three types: intronic lncRNAs (incRNAs), intergenic lncRNAs (lincRNAs), and antisense lncRNAs [66].

### Identification and function prediction of plant lncRNAs

There are two main steps to identify lncRNAs. First, the lncRNA transcript units are identified using high-throughput sequencing data or tiling microarrays data. Second, the coding potential of these lncRNA transcript units are calculated based on codon statistics, as well as the similarity to known protein-coding sequences. Several tools have been used to evaluate coding potential (Table 1), such as PhyloCSF [32], CPC [33], CPAT [35], and CNCI [34]. PhyloCSF uses the codon substitution frequency (CSF) score to distinguish protein-coding transcript units from non-coding ones. CPC and CNCI are developed based on support vector machine (SVM), whereas CPAT is built on the basis of a logistic regression model with sequence features. In addition, two standalone tools, iSeeRNA [37] and DeepLNC [36], have also been developed to identify lncRNAs from transcriptome sequencing data. iSeeRNA represents a computational pipeline to detect lncRNAs on the basis of SVM algorithm, whereas DeepLNC uses a deep neural network (DNN)-based classification model.

Previous studies suggest that lncRNAs perform different functions in various important biological processes. In order to determine the function and action mechanisms of lncRNAs, several tools have been proposed (Table 1). lncRNATargets [38] is used to predict lncRNA–mRNA or lncRNA–DNA interactions based on nucleic acid thermodynamics. spongeScan [39] can be used to predict lncRNA–miRNA interactions, thus identifying competitive endogenous RNAs (ceRNAs). In addition, the regulatory RNA motifs/functional sites and common transcription factors for a list of lncRNAs can be identified by RegRNA [41] and TF2LncRNA [40], respectively.

### Databases of plant lncRNAs

In order to organize the identified lncRNAs and their biological characteristics, several databases have been developed for further lncRNA research (Table 2). NONCODE 2016 [55] is a complete collection and annotation database of ncRNAs in 16 species, which covers various types of ncRNAs and evaluates lncRNA coding potential using CNCI software. However, among plant species, there are only Arabidopsis lncRNAs included in NONCODE. lncRNAdb [57] provides comprehensive annotations of 287 eukaryotic lncRNAs, as well as their experimentally-verified biological functions. Therefore, there are only few plant lncRNAs in lncRNAdb, *e.g.*, 7 Arabidopsis lncRNAs and 2 rice lncRNAs. Besides NONCODE and lncRNAdb, there are several plant-specific lncRNA databases available. Through integrating multi-omics datasets, such as tilling array, RNA-seq, epigenetic, and small RNA data, Jin et al. developed PlncDB, which provides comprehensive information related to lncRNAs, but only for Arabidopsis [56]. GREENC [56] is a Wiki-based database of plant lncRNAs, in which lncRNAs from 37 plant species were annotated, whereas PLNlncRbase [61] collects all experimentally-identified lncRNAs in 43 plant species. In addition, PNRD [59] and CANTATAdb [60] are the two largest plant lncRNAs databases. PNRD includes lncRNA sequences of mainly four plant species, including Arabidopsis, rice, poplar, and maize, while the recently-released CANTATA collects lncRNAs from 10 model plant species.

## Functional roles of ncRNAs in plant stress response

### miRNAs involved in stress response

As a post-transcriptional gene regulator, many miRNAs play key roles in stress response in plants, which have been the focus of several review articles published in recent years [67], [68], [69], [70], [71], [72].

**Figure 1:**
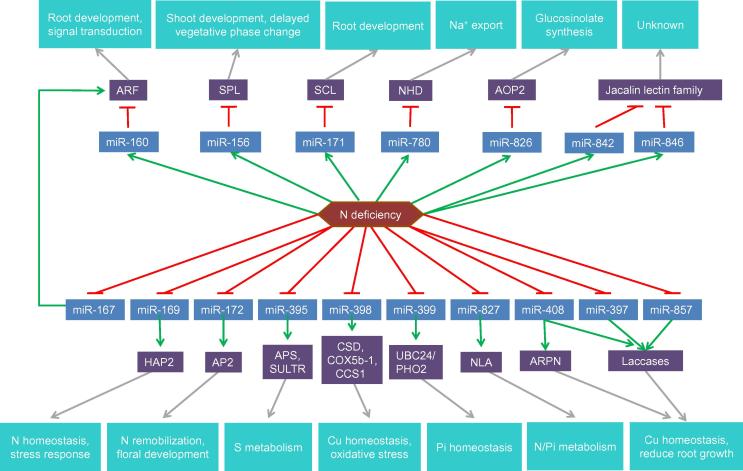
**miRNAs with altered expression during nutrient deficiency** Altered expression of miRNAs leads to a decrease (red blunt arrow) or increase (green arrow) in the expression of their target genes and corresponding proteins. ARF, auxin response factor; SPL, sporocyteless; SCL, scarecrow-like 3; NHD, Na^+^/H antiporter; AOP2, alkenyl hydroxalkyl producing 2; HAP2, heme activator protein 2; AP2, apetala 2; APS, ATP sulfurylase; SULTR, sulfate transporter; CSD, copper/zinc superoxide dismutase; cox5b-1, cytochrome C oxidase subunit 5b-1; CCS1, cytochrome C biogenesis protein; UBC24/PHO2, ubiquitin-conjugating Enzyme E2/phosphate 2; NLA, nitrogen limitation adaptation; ARPN, plantacyanin.

Similar to protein-coding genes, miRNA expression is up-regulated or down-regulated in response to stress (Figure 1). For example, in Arabidopsis, in response to nitrogen (N) deficiency, expression of miR-160 is upregulated whereas that of miR-169 is downregulated [73], [74]. Altered expression of these miRNAs is involved in the attenuation of plant growth and development during stresses. On one hand, N starvation induces the expression of miR-160, thus leading to a decrease in the expression of its target genes encoding auxin response factors (ARFs). ARF16 is reportedly involved in root cap cell formation, while ARF17 functions as a regulator of Gretchen Hagen3 (GH3)-like early auxin-response genes. The down-regulated expression of *ARF16* and *ARF17* would repress plant growth and thus increase tolerance to stress. On the other hand, the down-regulated expression of miR-169 leads to increased accumulation of its target gene family encoding nuclear factor Y (NFY), which binds to promoters of the genes encoding nitrate transporters, *AtNRT2.1* and *AtNRT1.1*, to regulate their expression [75].

**Figure 2:**
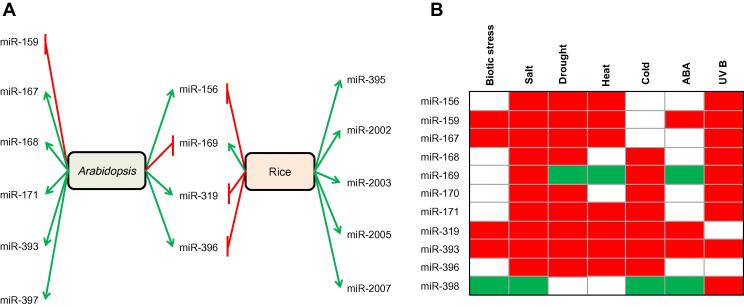
**Differential expression of miRNAs in stress response in plants** **A.** miRNAs commonly expressed in Arabidopsis and rice show the opposing expression patterns during heat stress. Expression of miR-156, miR-319, and miR-396 is up-regulated in Arabidopsis, but downregulated in rice, whereas expression of miR-169 was down-regulated in Arabidopsis, but up-regulated in rice. **B.** miRNAs are commonly involved in response to multiple stresses in Arabidopsis. Expression of miRNAs is either induced/increased (red), inhibited (green), or unaltered (white) under different stress conditions. ABA, abscisic acid; N, nitrogen; Pi, phosphate; S, sulfur; Cu, copper.

Some miRNAs in stress adaptation are conserved among plant species. For instance, several N deficiency-related miRNAs in Arabidopsis, such as miR-160, miR-169, miR-171, miR-395, miR-397, miR-398, miR-399, miR-408, and miR-827, were involved in response to N deficiency in maize [73], [76]. These miRNAs have similar expression pattern (upregulation of miR-160 and down-regulation of the others). However, opposing miRNA expression patterns have also been found in two different plant species during stress conditions (Figure 2A). In response to drought stress, expression of miR-156, miR-319, and miR-396 was induced in Arabidopsis, but inhibited in rice, whereas expression of miR-169 was down-regulated in Arabidopsis, but up-regulated in rice [77].

It is notable that some miRNAs are commonly involved in response to multiple stresses (Figure 2B). On one hand, expression of some miRNAs is induced or inhibited by multiple stresses. For example, in Arabidopsis, expression of miR-393 was found to be induced by at least seven types of stresses, whereas expression of miR-398 was inhibited by biotic stress, salt, cold, and abscisic acid (ABA) treatment. On the other hand, some miRNAs respond differentially to different stresses. For example, expression of miR-169 was induced by salt, cold, and UVB irradiation, but down-regulated during drought, heat, and ABA treatment in Arabidopsis [78]. Taken together, the aberrant expression of miRNAs during stress conditions is manifested in a stress-dependent manner. That is, the expression pattern of miRNAs relies on the specific stress condition.

Moreover, further studies have suggested that the aberrant expression of miRNAs has a tissue-dependent manner during stresses. Eldem and colleagues analyzed miRNAs expression profiles in root and leaf tissues subjected to drought. They found that in peach, drought induced aberrant expression of more miRNAs in root than in leaf tissue, suggesting root is more sensitive to drought than leaf tissue [79]. Furthermore, additional studies have reported that some miRNAs showed different expression patterns among different tissues during stress conditions. For instance, a recent study identified six wheat tissue-dependent miRNAs in response to drought stress, including miR-159, miR-172, miR-319, miR-399, miR-528, and miR-4393, whose expression was induced in leaves but inhibited in roots [79]. Similarly, Kantar and colleagues found four tissue-dependent miRNAs during drought [79]. Expression of miR-166 was induced in leaves but inhibited in roots, whereas expression of miR-156, miR-171, and miR-408 was up-regulated in leaves but unaltered in roots.

### The lncRNA-mediated stress response

**Figure 3:**
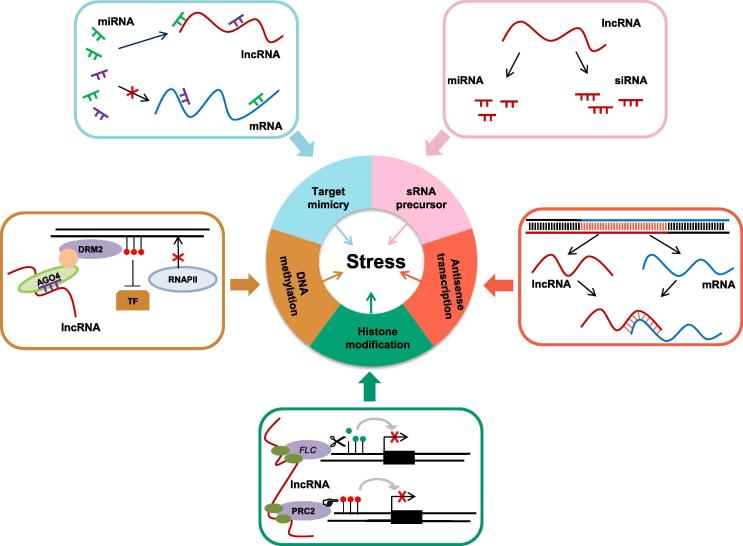
**Roles of lncRNAs in stress response in plants** The major mechanisms underlying the involvement of lncRNAs in stress response include target mimicry, sRNA precursors, NAT pairs, lncR2Epi, and RdDM. NAT pair, the interaction between sense mRNA and antisense lncRNA; lncR2Epi, lncRNA-mediated chromatin modification; RdDM, RNA-directed DNA methylation; FLC, FLOWERING LOCUS C (FLC); PRC2, polycomb repressive complex 2; DRM2, domains rearranged methyltransferase 2; AGO4, argonaute 4; TF, transcription factor; RNAPII, RNA polymerase II.

Recent studies have clearly suggested that lncRNAs, more than just transcriptional noise, can act as regulatory molecules in various development processes. Several reviews have given excellent summaries and discussions of the biological roles of plant lncRNAs [2], [3], [4], [5]. Moreover, emerging evidence has supported that lncRNAs are involved in stress responses. In the PLNlncRbase database, a total of 1060 stress-related lncRNAs were collected and documented under 17 different abiotic or biotic stress conditions in 43 plant species [61]. In plants, lncRNAs may execute their function to respond to stresses in five different ways (Figure 3).

First, some plant lncRNAs can act as ceRNAs, which are targeted by specific miRNAs via target mimicry, thus blocking the interactions between miRNAs and their targets. For example, in Arabidopsis, *IPS1*, whose expression is induced under phosphate (Pi) deficiency, acts as a target mimic for miR-399 and thus protects the miR-399 target gene encoding PHOSPHATE2 (PHO2), which is involved in Pi homeostasis via interacting with PHO1 [80]. In addition, by constructing ceRNA network, a recent study has revealed that lncRNAs compete with genes to play important roles in rice under Pi deficiency [6]. Similarly, several lncRNAs are identified as target mimics for tomato miRNAs in response to the infection of tomato yellow leaf curl virus (TYLCV) [81].

Second, certain plant lncRNAs can serve as sRNA precursors to produce sRNAs, such as miRNAs and siRNAs. In a recent study, 13 lncRNAs were predicted as precursors of 96 miRNAs involved in resistance to *Sclerotinia sclerotiorum* infection in *Brassica napus* [82]. In addition, 14 lncRNAs with precursor sequence for miRNAs were found in poplar under N deficiency, with nine lincRNAs as precursors for 11 known miRNAs and five for 14 novel miRNAs [8]. Another study in wheat reported three lncRNAs as precursors for miR-2004 and miR-2066, and 16 lncRNAs as precursors for 97 siRNAs responsive to powdery mildew infection [83].

Third, plant antisense lncRNAs are responsive to stress via the interactions with sense mRNAs. In our previous study, we constructed the PlantNATsDB, covering plant natural antisense transcripts (NATs) from 70 plant genomes [84], while our recent study showed that 828 lncRNAs took part in NAT pairs in Arabidopsis [85]. Antisense RNAs have been reported to be involved in gene expression regulation at multiple levels, including transcription, genomic imprinting, RNA stability, chromatin modification, transcription interference, and alternative splicing [86], [87]. Several investigations have suggested that antisense lncRNAs are involved in stress responses. For instance, five antisense lncRNAs were detected in poplar under N deficiency [8]. Similar results have also been reported in drought-stressed maize, stripe rust pathogen-infected wheat, or light-stressed Arabidopsis [88], [89], [90]. The antisense lncRNAs may form double-stranded RNA duplexes with sense mRNAs, thus affecting the gene expression on the opposite strand [90].

Forth, some plant lncRNAs are involved in lncRNA-mediated chromatin modifications (lncR2Epi) regulation pathway [91]. Expression of two cold-induced lncRNAs, *COOLAIR* and *COLDAIR*, has been reported to repress the expression of *FLOWERING LOCUS C* (*FLC*) through lncR2Epi regulation pathway in cold-stressed Arabidopsis [92], [93]. FLC is a MADS-box transcription factor, which can inhibit flowering under cold temperature [94]. On one hand, *COOLAIR* can mediate reduction in H3K36me3 or H3K4me2 at *FLC* [92], [95]. On the other hand, *COLDAIR* physically associates with polycomb repressive complex 2 (PRC2) to promote H3K27me3 accumulation at *FLC* [93].

The last, lncRNAs have a key role in RNA-directed DNA methylation (RdDM) pathway to stress response. 24-nt siRNAs and lncRNAs are both involved in the establishment of *de novo* cytosine methylation via the RdDM process. RdDM complex is directed by siRNAs produced from RNAPIV-dependent transcripts. Then these siRNAs load to Argonaute 4 (AGO4) to form silencing complex. Subsequently, RNAPII-dependent lncRNAs recruit AGO4 through interaction with AGO4-associated siRNAs. Ultimately, the AGO4–siRNA–lncRNA complex recruits DNA methyltransferase domains rearranged methyltransferase 2 (DRM2), resulting in *de novo* methylation of cytosine in all three sequence contexts (see reviews [96], [97], [98]). The RdDM pathway is suggested to be very important for biotic and abiotic stress tolerance. Down-regulated expression of RdDM pathway key factors significantly improved tolerance to stress. For instance, down-regulation of *SlAGO4A*, which encodes a core factor of RdDM pathway in tomato, significantly enhanced tolerance to salt and drought stress compared to wild-type and *SlAGO4A*-overexpressing plants [99]. A similar situation is observed for DNA methyltransferase 1 (MET1) [100].

Moreover, the mutants *ago4* and *nrpd2* (for the gene encoding DNA-directed RNA polymerases IV and V subunit 2) showed enhanced resistance to bacterial pathogen in Arabidopsis [101]. Similarly, Arabidopsis mutants *rdr2* for the gene encoding RNA-dependent RNA polymerase 2 and *dcl3* for the gene encoding Dicer-like 3 also showed reduction in survival rates in response to heat stress [102]. Some stress-related genes are activated more during stress conditions because of the loss of RdDM pathway. For example, expression of the gene At1g34220, encoding a regulator of vacuolar protein sorting 4 (Vps4) activity in the multivesicular body pathway, was induced by heat in wild-type Arabidopsis and had a higher level in *nrpd2* mutant [102]. A similar observation was obtained for myb domain protein 74 (MYB74) in response to salt stress [103]. These findings indicate that RdDM may be involved in DNA demethylase-mediated regulation of stress-response genes. However, expression of some stress-related genes is downregulated through the loss of RdDM pathway. For example, under normal situation, expression of *CARBON/NITROGEN INSENSITIVE 1* (*CNI1*) is induced, while expression of *CNI1-AS1*, transcribed in the antisense direction of overlap with *CNI1*, is inhibited by the methylation triggered by RdDM pathway [104]. During hyperosmotic stress, demethylation stimulates *CNI1-AS1* expression, which in turn causes the down-regulation of *CNI1* expression [104]. A similar result is also reported for *AT1G29450/COPIA*-like transposon AT1G29475 in response to heat stress [102]. Collectively, these results point toward an important role of RdDM pathway in stress tolerance.

## Conclusions and perspectives

miRNAs and lncRNAs are two important types of ncRNAs in plants, which play important roles in various biological processes. Rapid progress in high-throughput sequencing and advancement of bioinformatics tools provide revolutionary ways for identification and prediction of novel ncRNAs. In this review, we summarized the common bioinformatics tools and resource of miRNAs and lncRNAs in Tables 1 and 2. In addition, recently-developed single-cell sequencing and single-molecule sequencing will offer more opportunities to increase the number of ncRNAs. Therefore, it is necessary to develop new bioinformatics methods for the identification and functional analysis of ncRNAs from the single-cell or single molecule sequencing data.

Although remarkable progress has been made in explaining the role of plant miRNAs and lncRNAs in plant adaption to stress, mechanistic details are still limited. As described in this review, the mechanistic studies of ncRNAs involved in stress resistance are confined to a few cases (Figure 1, Figure 2, Figure 3). Therefore, more efforts are needed for systematic analysis of the regulatory roles and the involved pathways of ncRNAs in response to stress in future. It’s worth mentioning that the cross-talk between miRNAs and lncRNAs, lncRNAs as miRNA precursors [88], [89], [90] and ceRNAs [88], [89], [90], are the two major mechanisms of lncRNA-mediated stress response. In addition, previous studies have shown that alternate accumulation of miR-168 and miR-823 in response to fungal elicitors have an impact on RdDM pathway, as miR-168 and miR-823 target the genes encoding components of RdDM pathway, AGO1 and chromomethylase 3 (CMT3), respectively [105]. Thus, it would be interesting to investigate the cross-talk patterns between miRNAs and lncRNAs and construct the cross-talk network, which will expand our knowledge on gene regulation networks in stress response.

While our review focuses solely on the roles of miRNAs and lncRNAs in stress response, accumulating evidence also points out that siRNAs are involved in plant stress adaptation. Plant siRNAs can be further categorized as NAT siRNAs (nat-siRNAs), heterochromatic siRNAs (hc-siRNAs), and secondary siRNAs including *trans*-acting siRNAs (ta-siRNAs) [106]. nat-siRNA has been reported to function in salt-stressed Arabidopsis, representing the first line of evidence showing the involvement of siRNAs in plant stress responses [107]. In Arabidopsis, the gene encoding delta1-pyrroline-5-carboxylate dehydrogenase (*P5CDH*) overlaps with the gene encoding similar to rcd one 5 (*SRO5*) in antisense orientation. *P5CDH* is constitutively expressed, whereas expression of *SRO5* is induced by salt stress. Under salt stress condition, *SRO5* can produce a 24-nt nat-siRNA, which mediates the cleavage of *P5CDH* transcript. The down-regulated expression of *P5CDH* by the 24-nt nat-siRNA reduced the proline degradation and enhanced proline accumulation [108]. The high level of proline is a positive factor for salt tolerance [109]. Notably, AGO4-loaded hc-siRNAs are recruited by nascent RNAPV transcripts and then guide RdDM pathway [110]. Microarray analysis has revealed that siRNA415, an hc-siRNA, accumulated abnormally in response to fungal elicitors, although siRNA415 guides DNA methylation at still unknown genomic loci [105]. In addition, expression of 12 ta-siRNAs and two nat-siRNAs was reported to change significantly under chilling stresses in cassava [111]. However, the mechanisms of siRNAs in regulating gene expression need further investigation, and experimental validation becomes an important step before making conclusions on the biological functions of the identified siRNAs.

Compared with the roles of sRNAs and lncRNAs in stress response, little was known on medium-sized ncRNAs. Recently, sRNAs arising from medium-sized RNAs, tRNAs (75–87 nt), and small nucleolar RNAs (snoRNAs, ∼150 nt) have been detected [112], [113], [114]. The production of tRNA-derived sRNA (stRNAs/tRFs) and snoRNA-derived sRNAs (sdRNAs) is induced by various stresses. Previous studies have shown that phosphate deprivation and drought stress trigger the cleavage of specific tRNAs in Arabidopsis and barley. In addition, stRNAs have been predicted to be heat-responsive in *Brassica rapa* [115] and wheat [116]. The up-regulation of stRNAs during virus or bacterial infection such as apple stem grooving virus (ASGV) infection [117] and *Phytophthora capsici* infection [118] was also reported in plants. The next-generation sequencing technologies also provided evidence that sdRNAs are involved in the heat response in wheat [116]. Although the biological function mechanisms of stRNAs and sdRNAs are not yet clear, they may be involved in the inhibition of protein translation or regulatory mechanism like siRNAs or miRNAs [119]. Moreover, although snoRNAs can work as RNA chaperones to aid the folding of other RNAs [120], there is no scientific evidence showing the relationship between snoRNAs as RNA chaperones and stress response.

With the accumulation of data, it will be exciting to witness the further evolution of our knowledge on the functional roles of ncRNAs in stress response.

## Competing interests

The authors have declared no competing interests.
